# Supplementary material for: Novel molecular requirements for CRISPR RNA-guided transposition
Source: Nucleic Acids Res. 2023 Apr 20;51(9):4519–35. doi: 10.1093/nar/gkad270 (PMC10201428; doi:10.1093/nar/gkad270)
Supplement: gkad270_Supplemental_Files [file gkad270_supplemental_files.zip › Supplementary_Figures.pdf]

SUPPLEMENTARY FIGURES

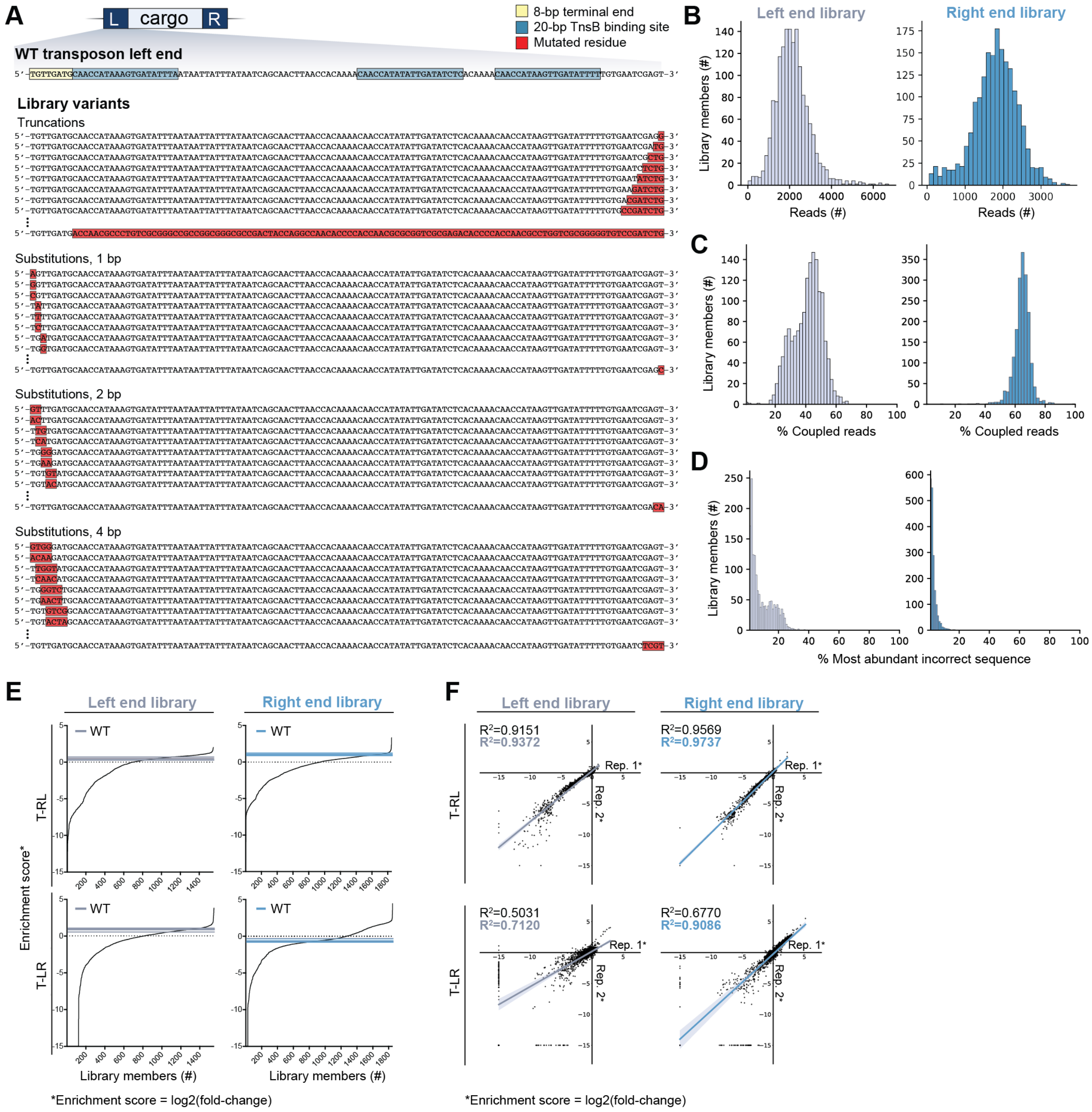

Figure S1. Sequencing and characterization of pDonor right end and left end pooled libraries. (**A**) Schematic of select transposon left end library variants. Red shading indicates residue(s) that are mutated in the library variant. (**B**) Histogram showing read counts for each of the input libraries, as defined by barcode sequences. All library members are represented in the transposon left end and right end libraries. (**C**) Histogram showing the percentage of each library member's high-quality reads in which the correct barcode is coupled to the correct transposon end sequence. Library members are identified by their barcodes. (**D**) Histogram showing the highest percentage of each library member's uncoupled reads

mapping to a single incorrect sequence. In other words, for a given library member, we selected the incorrect (uncoupled) sequence with the highest read count and expressed that read count as the percent of total reads for that library member. These analyses demonstrate that only a small minority of all barcode reads for a given library member are associated with an incorrect (uncoupled) transposon end sequence. (**E**) All enrichment scores for library members in either integration orientation, for both the left end and right end libraries. Enrichment scores were calculated by dividing the abundance of each member in the output library by its abundance in the input library, and then taking the log2 transformation of that value. Library member dropouts were arbitrarily assigned a score of -15, which fell below the minimum enrichment score across all samples, in order to be plotted on the same graphs. Enrichment scores for WT library members, represented by four different barcodes, are highlighted and indicate consistency of the library results. (**F**) Correlation between two independent biological replicates for the transposon left and right end library transposition experiments. For each graph, the upper R2 value (black) includes enrichment scores for all transposon end variants, where dropouts were arbitrarily set to -15. The lower R2 value (colored) includes only the enrichment scores for transposon end variants that were detected in both output libraries.

Figure S2

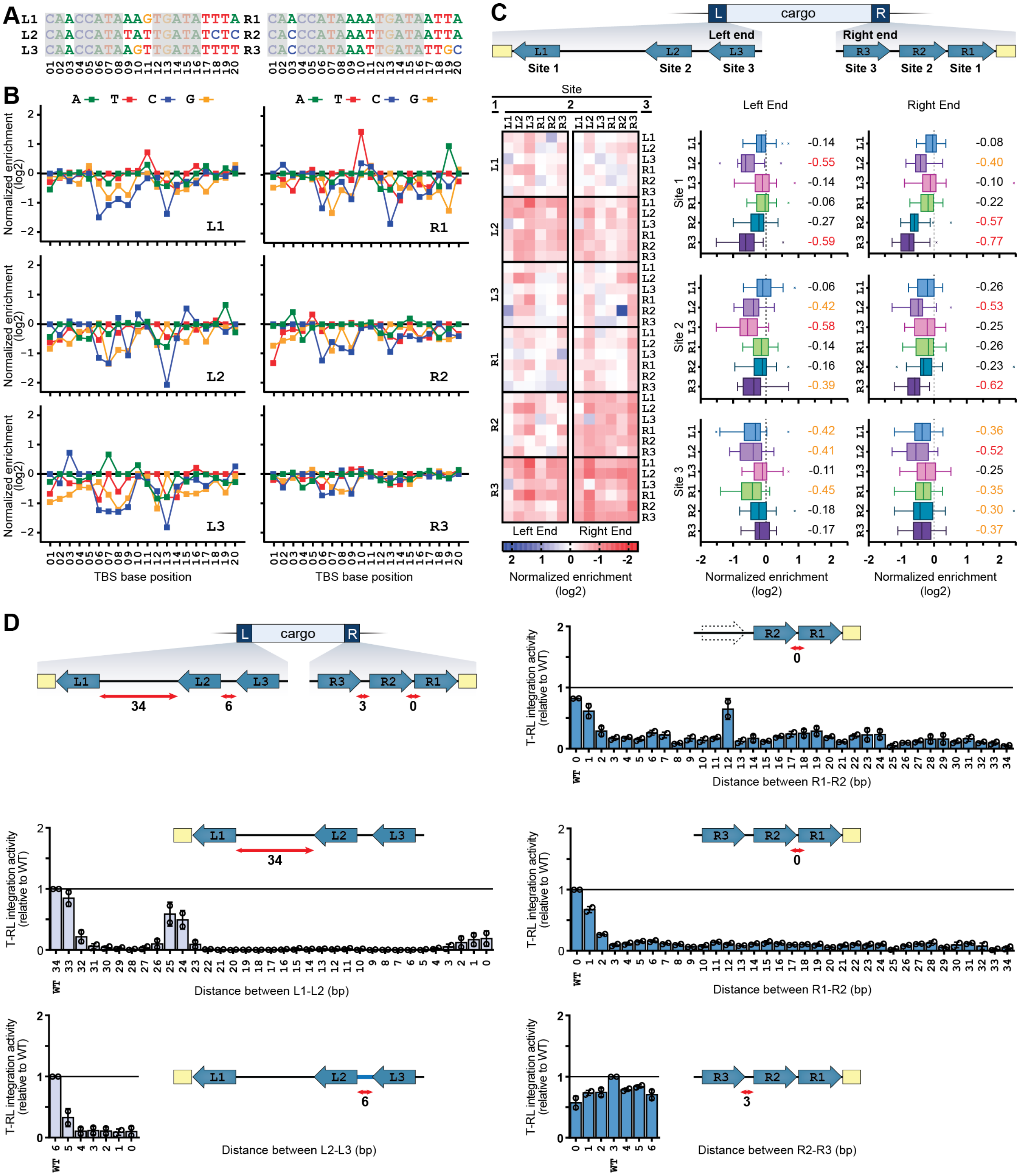

Figure S2. Sequence and spatial requirements of VchCAST TBSs. (**A**) Sequence conservation among the six bioinformatically predicted TBS sequences, with nucleotides conserved among all six sites highlighted in gray. (**B**) Integration activity for mutagenized TBS sequences at individual binding sites, shown as the mean of two biological replicates. Integration activity is represented as the library variant enrichment score

normalized to WT. (**C**) Schematic representation of the transposon end architecture (top). Enrichment of individual transposon end variants for which the TBS were shuffled are shown as a heatmap (left). The overall effect of each TBS is represented in a boxplot for the individual sites within both the left and right transposon ends, including their numerical mean (right). (**D**) Schematic representation of the spacing in between the TBS sequences of the transposon left and right ends (top left). Red arrows indicate regions where the distance between either individual binding sites or groups of binding sites was modulated. Integration efficiencies, calculated from enrichments within the larger transposon end library dataset, are shown for alternative spacing between the TBS sequences of the left and right end sequences.

Figure S3

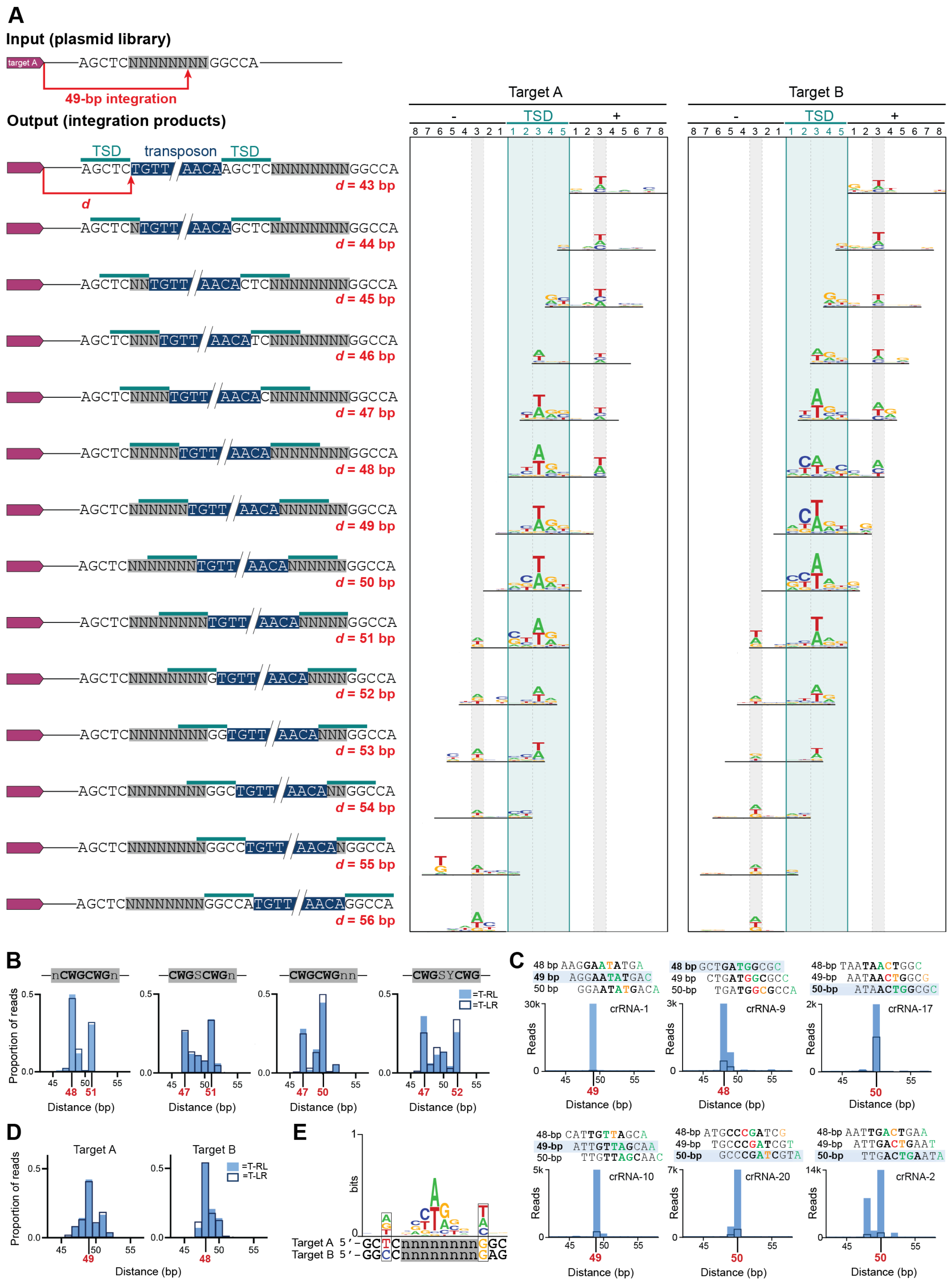
A
Input (plasmid library)
49-bp integration
Output (integration products)
TSD
transposon
Target A
Target B
B
Proportion of reads
Distance (bp)
=T-RL
=T-LR
C
Reads
crRNA-1
crRNA-9
crRNA-17
crRNA-10
crRNA-20
crRNA-2
D
E
bits

Figure S3. Transposase sequence preferences at the site of DNA integration. (**A**) Schematic of Target A integration products, with corresponding sequence logos of enriched sequences at each integration position. Sequence logos were generated by selecting all sequences with 4-fold enrichment in the integrated products compared to the input libraries. The y-axis of each sequence logo was set to a maximum of 1 bit. (**B**) Integration site distance distribution for degenerate sequences containing multiple preferred CWG motifs, with preferred distances indicated in red. (**C**) Integration site distance distributions of previously tested genomic target sites, as determined through deep sequencing. The TSD sequence ±3-bp is shown for distances of 48, 49, and 50 bp. Integration occurs primarily 49-bp downstream of the target site but can be biased to occur 48- and/or 50-bp downstream due to sequence preferences at the site of integration. The TSD is bold, and favored (green) or disfavored (orange and red) nucleotides according to the preference sequence logo are indicated. (**D**) Integration site distance distribution for two targets, A and B, with preferred distances indicated in red. (**E**) Nucleotide preferences surrounding the degenerate sequence may be responsible for differences in the overall integration site distance distribution.

Figure S4

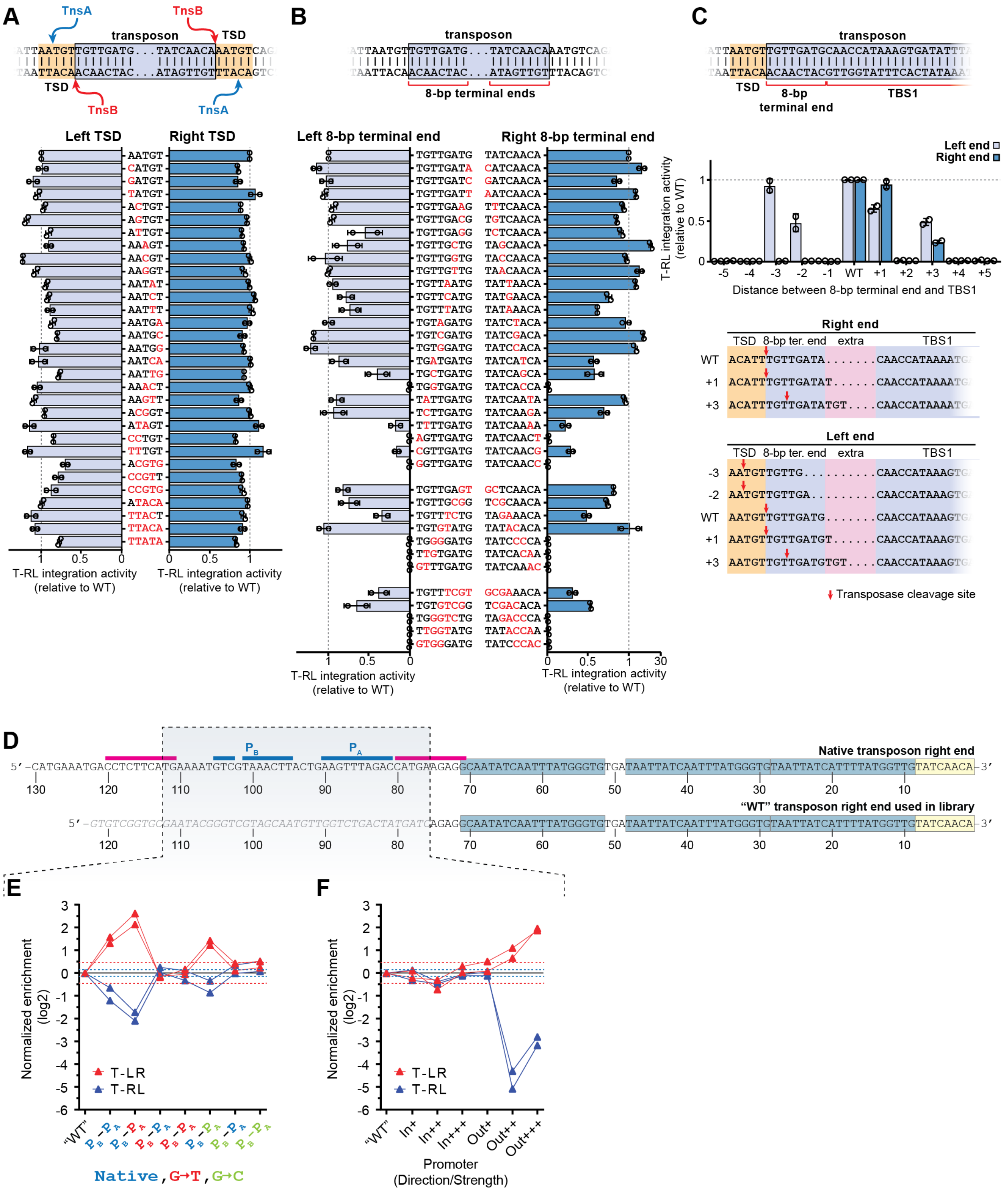

Figure S4. Effect of target-transposon boundary sequences and internal sequences on DNA integration. (**A**) Schematic representation of DNA cleavage by TnsA and TnsB, leading to full excision of the transposon from the donor site (top). Different transposon-flanking sequences were tested on both the left

and right transposon boundaries, and integration efficiencies were determined by calculating the enrichment of each library member from within the larger transposon end pool (bottom). (**B**) Illustration of the imperfect 8-bp terminal end sequences for VchCAST (top). Calculated integration efficiencies are plotted for transposon end variants in which either the left or right terminal end sequence was mutated. (**C**) Illustration of the transposon end sequences including the target site duplication (TSD), 8-bp terminal end, and first transposase binding site (TBS1). The specific sequence shown is derived from the VchCAST left end (top). Integration efficiencies relative to WT are shown for transposon end variants in which the distance between the 8-bp terminal end and TBS1 was altered for either the transposon left or right end (middle). Analysis of deep sequencing data revealed TnsB cleavage sites for the right end and left end variants that were functional for transposition; cleavage sites are indicated with red arrows. (**D**) An illustration of native and library variant transposon right end sequences. The 8-bp terminal end (yellow boxes), transposase binding sites (blue boxes), and palindromic sequences (blue and pink lines), are indicated. The native sequence encompasses 130 bp from *V. cholerae* Tn*6677*, whereas only 75 bp were used in the "WT" sequence used in library experiments. (**E**) Integration activity of right end library variants, in which the palindromic sequence was altered. Integration activity is represented as the library variant enrichment score normalized to WT. Each variant included a distinct combination of palindromic sequences P_B and $P_{A,,}$ with the ordering as shown. Blue text ("native") indicates the native palindromic sequence. Orange text ("G-T") refers to variants in which palindrome nucleotides were mutated from G to T and A to C. Green text ("G-C") refers to variants in which palindrome nucleotides were mutated from G to C and A to T. (**F**) Integration efficiencies of right end variants in which different internal promoter sequences point inwards of the transposon (In) or outwards across the transposon end (Out). Promoter strengths are indicated pJ23114 (+), pJ23111 (++), pJ23119 (+++).

Figure S5

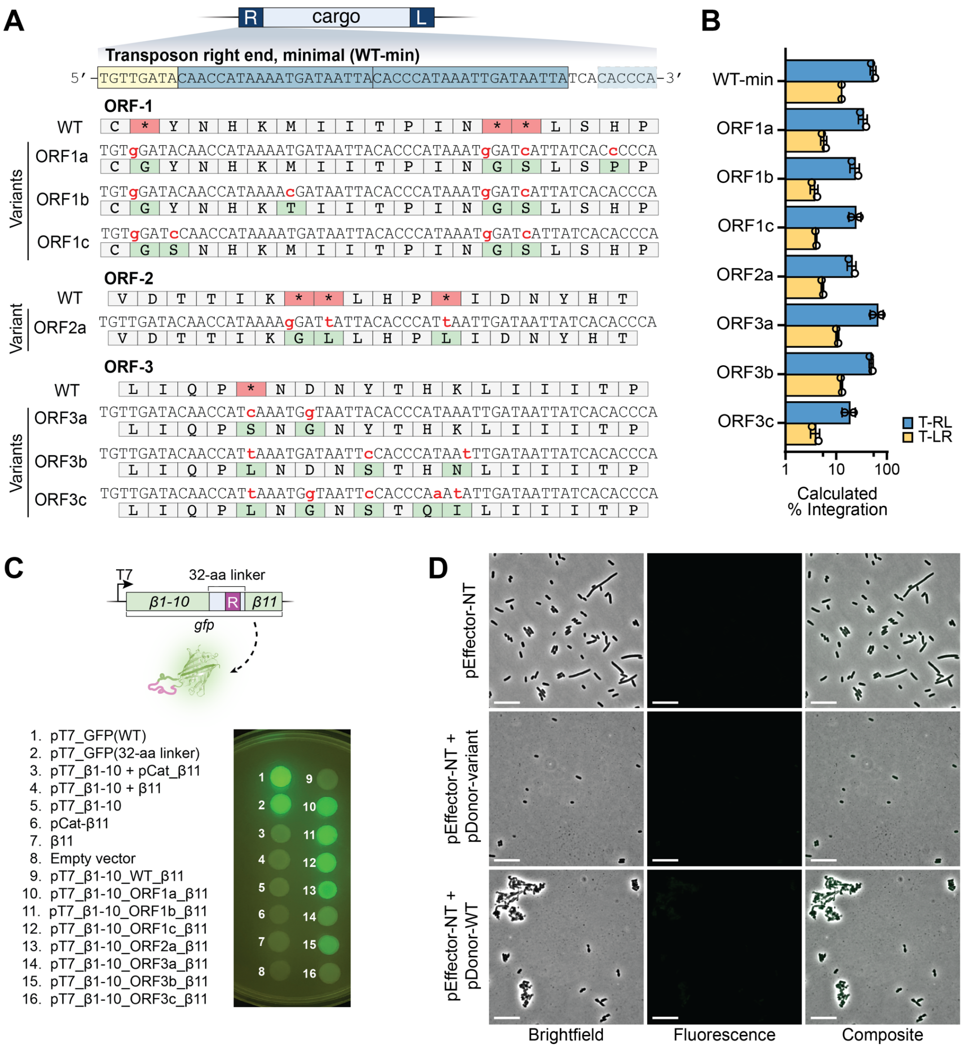

Figure S5. Engineering of the VchCAST right end. (**A**) Schematic of transposon right end linker variants. Green shading indicates amino acids that differ from the WT ORF. (**B**) Integration data for transposon right end variants that were modified to encode functional protein linker sequences in each of three open reading frames (ORF1–3). Integration efficiencies were calculated based on enrichment values within the library dataset. (**C**) Schematic representation of the linker functionality assay in which GFP includes a linker sequence encoded by a mutated right end (top). The fluorescence of *E. coli* cells expressing each of the indicated GFP constructs was visualized upon excitation with blue light (bottom). (**D**) Fluorescence microscopy images of negative control samples for the C-terminal GFP-tagging experiment, showing a brightfield image (left), fluorescence image (center), and composite merge (right). Controls included experiments testing a non-targeting pEffector alone (top) or in combination with either a transposon encoding a functional linker variant (middle) or a wildtype transposon (bottom). Scale bar represents 10 μm.

Figure S6

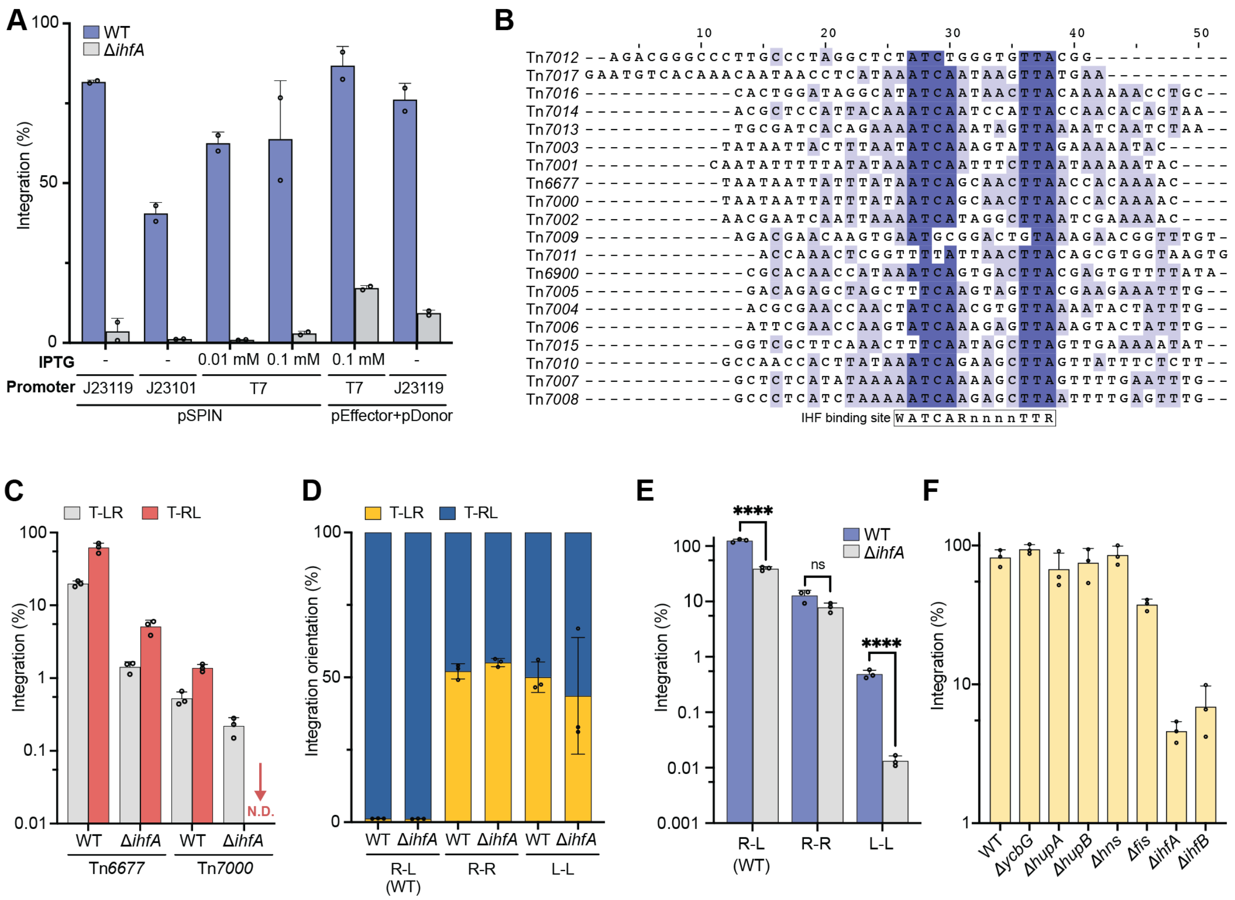

Figure S6. Transposition efficiency of VchCAST and other Type I-F CAST systems in WT and NAP-knockout cells. (**A**) Integration efficiency under different expression systems and induction conditions for VchCAST in WT and Δ*ihfA* cells. pSPIN is a single plasmid that encodes both the donor molecule and transposition machinery, as described in (33). pEffector+pDonor refers to separate plasmids that encode the transposition machinery and donor DNA, respectively. The indicated promoters were also tested, with J23119 and J23101 being constitutively active whereas the T7 promoter is induced by growing cells on IPTG. (**B**) Alignment of the sequence between the first two TnsB binding sites (L1 and L2) in the left end, generated by Clustal Omega and colored in Jalview to highlight conserved residues. The consensus IHF binding site is shown below the alignment. (**C**) Integration orientation preference in WT and Δ*ihfA* cells for VchCAST and Tn*7000*. For Tn*7000*, T-RL integration products were not detected (N.D.) after 35 cycles of qPCR, indicating an integration efficiency less than 0.01%. (**D,E**) Integration orientation (**D**) and efficiency (**E**) of transposons with symmetric end sequences in WT and Δ*ihfA* cells. R-L refers to a WT-like sequence in which the transposon end identity has not been changed, whereas R-R or L-L refer to transposons in which the left or right end sequence have been mutated to the opposite end sequence, resulting in a transposon with symmetric ends. **F**) Effect of nucleoid associated protein knockouts for

VchCAST. Transposition was measured by qPCR after expressing pSPIN in each of the indicated *E. coli* knockout strains.

Figure S7

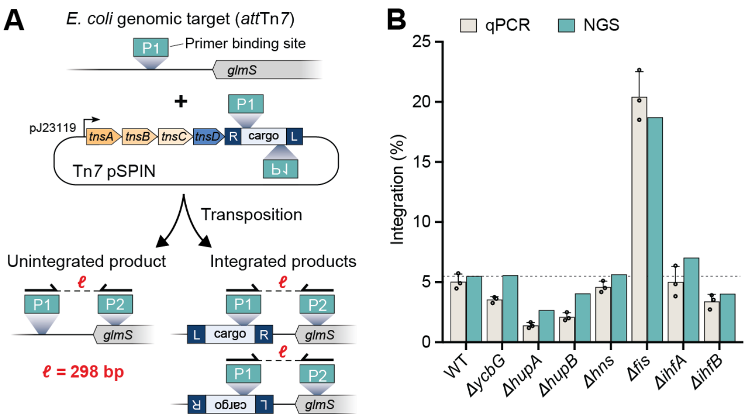

Figure S7. Effect of NAP knockouts on *E. coli* Tn*7* transposition efficiency and fidelity. (**A**) Schematic of NGS-based Tn*7* transposition assay. The transposon cargo encodes genomic primer binding sites ("P1") adjacent to the right and left ends, such that the NGS amplicon length ("ℓ") is the same for unintegrated products and for integrated products in both orientations. Using this strategy, a single NGS library reports both the integrated and unintegrated products, while avoiding PCR bias that might arise from amplifying products of different lengths or primer binding sites. (**B**) Tn*7* integration efficiencies in the indicated NAP knockout strains are shown, quantified using both qPCR and NGS. The dotted line shows the WT integration value as measured by NGS. Δ*ihfA* or Δ*ihfB* have no effect on integration activity, whereas Δ*fis* increases integration activity ~4-fold. (**C**) Integration distance and orientation distribution downstream of the *glmS* locus for Tn*7* in WT and Δ*fis* cells. The x-axis refers to the distance in bp between the stop codon of *glmS* and the integration site. For WT and knockout cells, the dominant distance is the canonical 25 bp downstream of *glmS*. The y-axes are shown as linear scale (top) and as log10 scale (bottom), in order to highlight low frequency integration events at non-canonical distances and orientations.
